# When workaholism is negatively associated with burnout: A moderated mediation

**DOI:** 10.3389/fpubh.2022.968837

**Published:** 2022-10-25

**Authors:** Irsa Fatima Makhdoom, Najma Iqbal Malik, Mohsin Atta, Nudra Malik, Madeeha Gohar Qureshi, Muhammad Shahid, Kun Tang

**Affiliations:** ^1^Department of Psychology, University of Sargodha, Sargodha, Pakistan; ^2^Department of Applied Psychology, Lahore College for Women University, Lahore, Pakistan; ^3^Department of Economics, Pakistan Institute of Development Economics, Islamabad, Pakistan; ^4^School of Insurance and Economics, University of International Business and Economics, Beijing, China; ^5^Vanke School of Public Health, Tsinghua University, Beijing, China

**Keywords:** burnout, cognitive demands, PsyCap, social load, time pressure, university teachers, workaholism, moderated mediation

## Abstract

**Aim:**

Previous theory and research postulate that workaholism is one of the important factors that contribute to burnout. The present study aimed to analyze the role of psychological capital as a mediator between the two. Moreover, the study examined the moderating role in the stated mediated relationship.

**Methods:**

The researchers approached a sample of university teachers (*N* = 1,008) including both male (*n* = 531) and female (*n* = 477) university teachers by using a multi-stage random sampling technique. For this purpose, DUWAS-10 Oldenburg Burnout Inventory, Challenging Job Demands Scale, and Anila PsyCap Scale were applied to measure workaholism, burnout, challenging job demands, and PsyCap, respectively. The data obtained from the sample was subjected to analysis by using Model 14 of Process Macro by Hayes.

**Results:**

The results confirmed the mediating role of PsyCap and moderating role of time pressure and cognitive demands in the relationship of the two variables. The results concluded that workaholism is not directly related to burnout rather the negative relationship existed through psychological capital, and the mediated relationship was stronger for the university employees who were to face a higher level of challenging job demands including cognitive demands and time pressure.

**Conclusion:**

Burnout is an occupational health problem that causes devastating effects on both the employees as well as to the organizational economy. Improving personal resources might help the negative relationship between workaholism and burnout in higher education institutions in the country.

## Introduction

Burnout, a stress-related outcome (1–4), is one of the most serious occupational health-related problems. Every year in the United States only, 120,000 employees die out of it, and thus, it leads to 190 billion dollars being spent by employers. Further, burnout is strongly and positively related to other mental and physical health illnesses such as depression and anxiety which results in a productivity loss of one trillion dollars annually (5, 6). Similarly, a research report (7) concluded that it costs between 1 to 2 billion dollars in lost revenue in the veterinary industry. Such statistics suggest the importance of burnout in the world economy.

Although several theories have been proposed to explain the processes behind burnout, Job Demands/Resources Theory (2) is one of the most welcomed theories among burnout researchers that initially outlined that there were certain job demands which affect the employee negatively and thus is bad for employees' health and motivation. These bad things were termed job demands. Equally, there were certainly good things that could reduce the effects of job demands, brought positivity to employees' health, and added to their motivation. These were labeled as job resources. Later, researchers found that not all job demands brought negative consequences. Rather some job demands, besides being stressful and thus affecting the health of the employees, brought some positive consequences also, for example, personal growth, feeling of mastery, and competence. This difference resulted in two forms of job demands i.e., challenging job demands and hindering job demands.

The current study includes three job demands as challenging that has been outlined in the indigenous culture of Pakistan in a sample of University teachers (3). Afterward, a ratio of job demands and resources was identified which stated that an increase in ratio resulted in an increased level of health and productivity (8). On the conceptual level, later researchers found that along with organizational resources, certain individual differences performed as resources and acted in the same way as organizational resources did. Such individual differences were labeled as personal resources (9, 10). Such personal characteristics often act as third variables in the demands-outcome relationship (11). For the present study, the researchers have focused on the personal resource of psychological capital.

Along with demands and resources, the researchers explained other work-related behaviors and outcomes such as innovative behavior and personal demands through the JD-R model (12, 13). One such construct is workaholism. Workaholism is traditionally defined as an addiction to work. It is characterized by excessive working and working out of some inner compulsion, and is marked by an obsession to work without any external incentive such as financial gain or monetary rewards, etc (14). Ostensibly, it seems that the construct is positive and may result in positive outcomes for both the employee and the organization, however, the studies have found mixed results regarding its effects. The previous research concluded both positive and negative correlates of workaholism. Among the negative outcomes, there are deteriorated employees' health, increased stress, more perpetration of CWBs, increased work-family conflict, and marital dissatisfaction. Similarly, it often results in increased burnout (14–16). However, work enjoyment, organizational commitment, and job involvement are those factors that are found positively associated with workaholism. Interestingly performance is found to be uncorrelated with overall job performance (17). Hence, more roles of workaholism in the JD-R Model are yet to be explored. Therefore, the purpose of the present research includes (1) to find out if personal resources (i.e., PsyCap) link workaholism and burnout, and (2) to explore whether the relationship between workaholism and burnout through the personal resource (i.e., PsyCap) changes as a function of challenging job demands (i.e., time pressure, social load, and cognitive demands).

### Workaholism and burnout

Traditionally, workaholism and burnout are supposed to positively relate to each other. Since workaholism involves excessive and compulsive thinking about work and thinking of work even when not working, it may eventually result in depleted emotional resources. This depletion of emotional resources, according to Conservation of Resources Theory (18), may result in the experience of job burnout. Moreover, workaholics spend more hours at work, they find lesser time to regain their depleted emotional resources, and thus are at greater risk of developing symptoms of burnout (19). Therefore, a positive relationship between the two is expected. Several researchers have concluded the same. For example, Cheung and colleagues (20) found that workaholism and burnout are positively related and that the relationship was very strong and was the same across different cultures and countries. Similarly, Schaufeli et al. (1) observed that workaholism resulted in more role conflicts which eventually lead to a higher level of burnout among employees.

On the other hand, workaholism may bring some positive outcomes too. For instance, it can beget promotions and managerial status (21) as well as positively relate to personal accomplishments and satisfaction with the leadership components of burnout (22). The mixed findings regarding its effects on burnout and other work-related positive and negative outcomes give room to search for certain third variables and the mechanism through which the relationship operates. Although much work has been done to explain the mechanism through which workaholism relates to burnout and other work-related outcomes, the complete mechanism is yet to be explored. Role conflict (23) and work-family conflict (24) are often researched mediators for the workaholism-outcome relationship. However, both of them explain the positive relationship between workaholism and burnout. There is a need to explore how workaholism negatively relates to burnout or which possible third variables could relate workaholism to burnout negatively. We propose that personal resources (i.e., PsyCap) and challenging job demands (i.e., time pressure, cognitive demands, and social load) may affect this relationship.

### Workaholism, psychological capital and burnout

The present study assumes that the relationship of workaholism with health-related outcomes (i.e., job burnout) is not simple; rather, the relationship is complex and may be affected by certain third variables. The researchers suggest that personal resources and challenging job demands act as third variables in the model. Among other personal resources, psychological capital (PsyCap) is often considered an important personal resource. PsyCap is defined as a strong mental state which is characterized by personal growth and is marked by hope, resilience, self-efficacy, and optimism (25, 26). Although being a workaholic does not guarantee increased performance, spending more hours at work simply may result in more work done. Similarly, it is positively associated with eustress (27) which may help employees to take work tasks as challenges, and thus they might feel grown. Therefore, as previous literature states, workaholism positively predicts PsyCap and its all four sub-scales including hope, resilience, self-efficacy, and optimism (28). The same positive relationship with workaholism has been found by previous researchers (29). Workaholism when accompanied by an external locus of control, may act as a resilience capacity or at even a higher level as learned resourcefulness (30).

On the other hand, PsyCap has often been attributed as a personal resource in the literature on JD-R Model. Since PsyCap includes feelings of mastery, hope and motivation for the future, and a feeling of personal capability in the form of self-efficacy, it may directly affect burnout. Particularly, the resilience component was a strong negative predictor of burnout in previous literature (31). Moreover, it may affect the perceptions of other demands and resources and the employee who is high at PsyCap might take these demands and resources as more positive to them (32, 33) and therefore, it is found as a negative predictor of burnout. Simply stated, the employee who expects positivity for the future, takes a brighter look at every aspect, or easily come back to routine after setbacks, and believes that s/he can complete the tasks (i.e., who is high in four components of PsyCap) will experience lesser stress and will be less affected by negative outcomes of stress. Therefore, PsyCap can act as a protective factor against developing the symptoms of burnout directly and through positive factors for example flourishing and positive coping, etc., (34–36). Therefore, we propose that workaholism will positively affect PsyCap which in turn would negatively affect burnout. Therefore, we hypothesize that.

H1: Psychological capital mediates the relationship between workaholism and burnout.

### Challenging job demands as moderators in the relationship of psychological capital and burnout

Challenging job demands, according to the Job Demands/Resource Model, are those job demands or stressors which, besides being negative and stressful, also contribute to the achievement and personal growth of the employees (37). Examples of such job demands are time pressure, cognitive demands, and workload (38). Although a considerable amount of research has been carried out about the differential effects of challenging and hindering job demands on work engagement and burnout, more rigorous evidence is required to clarify the various differential roles these job demands play (39). For the present research, three challenging job demands were studied including time pressure, cognitive job demands, and social load. Time pressure was defined as the pressure that is exerted when the job tasks have to be done using lesser time than what is available. It is the stress which results from the conflict of lesser time and more responsibilities to be completed. Cognitive job demands include those job demands which need higher-order cognitive functioning to complete the task. Finally, there is the social load. The social load includes those job demands which occur with the interaction of people at work. The three are found as challenging job demands in literature (40–42).

These job demands result in many positive outcomes including increased motivation, satisfaction, performance, and overall well-being of the employees (37, 43). Apart from its direct and clearer effect, the role of challenging job demands is more complex than it seems to be. The researchers have argued that not the nature of job demands (i.e., either challenging or hindering) but the appraisals associated with these job demands actually result in either favorable or unfavorable outcomes; and even the appraisal is not solely responsible for the particular outcomes, as the degree of these demands also matters. They say that no matter how the demands are appraised, some job demands can start acting as hindrances after a certain degree and level (44).

Therefore, the job demands can act as a challenge for one group of the population and as a hindrance for another. Similarly, they can act as a challenge up to a certain level and degree. However, apart from outcomes based on their appraisal, the challenging job demands may also play a role while interacting with other variables. For instance, time pressure works best when satisfaction with work-life balance is there or the quality of leader-member exchange is high (45). Similarly, when professionals (such as nurses) had to face time pressure along with other factors such as poor sleep, they were more likely to give false medicines and were more likely to accidentally injure their patients (46). However, little is known about the interactive effects of other challenging job demands (i.e., cognitive demands and social load). One study (47) found that cognitive demands and cognitive resources both interact with each other to result in higher professional efficacy in a sample of informatics. Even lesser data is available for the role of social demands in the stated variables. However, based on the proposition that challenging job demands act as positive agents for the employees like university teachers, they will bring positive outcomes in the relationship between PsyCap and burnout. More specifically, we propose that the negative relationship between PsyCap and burnout will be strengthened when there are highly challenging job demands. Therefore, the hypothesis of the study is:

H2: Challenging job demands moderate the strength of the indirect relationship between workaholism and burnout in university faculty *via* psychological capital as the indirect relationship is strengthened when challenging job demands in respondents are high.

H2(a): Time pressure moderates the strength of the indirect relationship between workaholism and burnout in university faculty *via* psychological capital such as the indirect relationship is strengthened when time pressure is high.

H2(b): Social load moderates the strength of the indirect relationship between workaholism and burnout in university faculty *via* psychological capital such as the indirect relationship is strengthened when a social load is high.

H3(c): Cognitive demands moderate the strength of the indirect relationship between workaholism and burnout in university faculty *via* psychological capital such as the mediated relationship is strengthened when cognitive demands are high.

**Figure 1:**
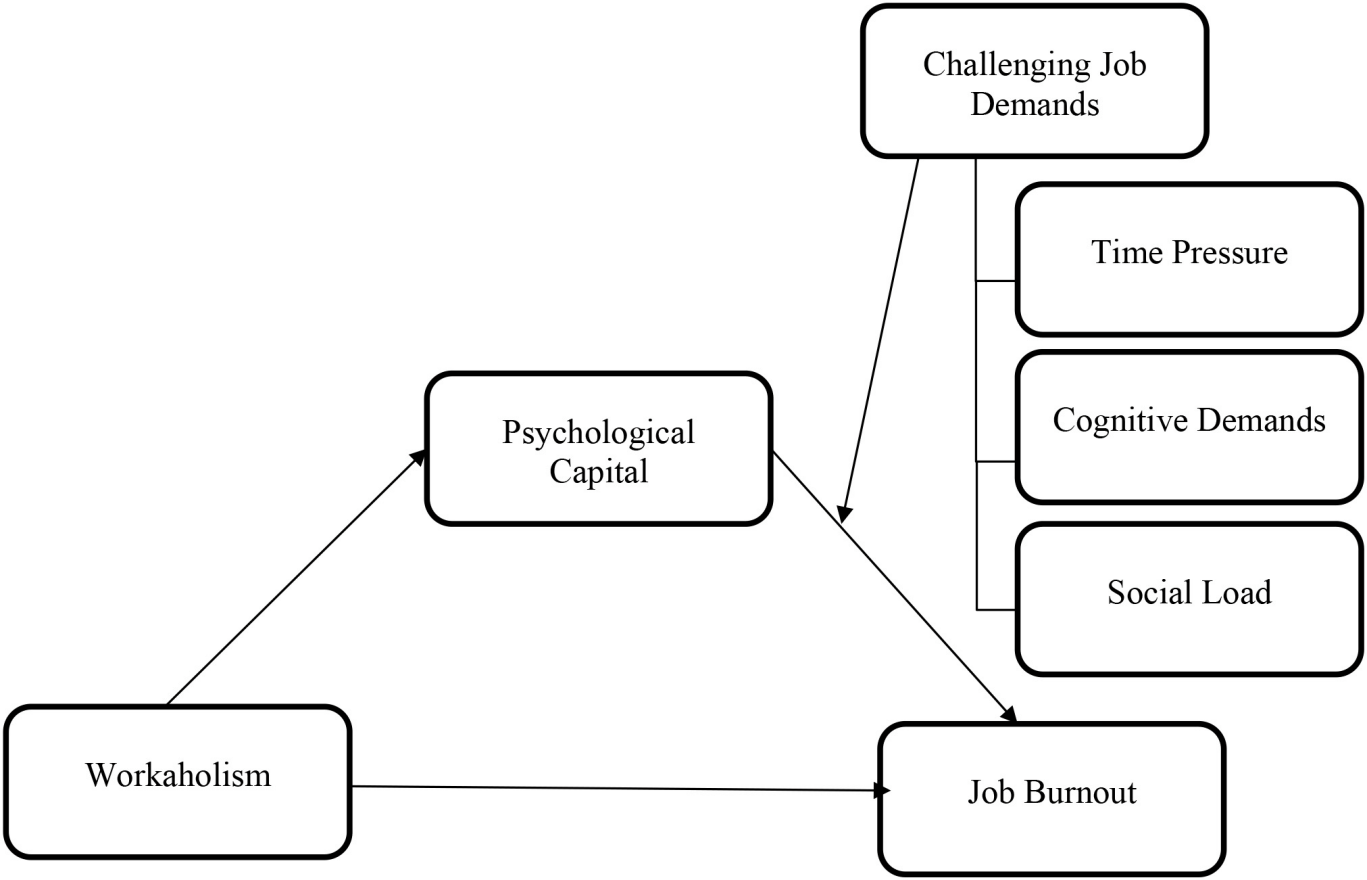
Proposed model of the study.

The conceptual framework of the study (see Figure 1) suggests that workaholism is a predictor of burnout and that workaholism and burnout are related through psychological capital. Moreover, challenging job demands would affect this mediated relationship. Therefore, the main purpose of the research is to find out the relationship of workaholism and burnout through PsyCap and also to explain if the mediated relationship differs in terms of different levels of challenging job demands (i.e., time pressure, cognitive demands, and social load).

## Methods

The present study followed a correlational research design where a survey was used as a research method.

### Participants

The sample of the present study included teachers (*N* = 1008) from different public sector universities in Pakistan through multi-stage random sampling. The sample size was determined through G-Power analysis using α (0.05) and a small effect size (0.02) with five independent variables. The sample size estimated through G-Power analysis was *N* = 646. Out of eight administrative units of Pakistan (including Islamabad, Punjab, Sindh, Khyber Pakhtunkhwa, Balochistan, Gilgit Baltistan, Azad Jammu and Kashmir, and Federally Administered Tribal Areas), the equal number of university teachers (*n* = 252) from four areas including Islamabad, Punjab, Khyber Pakhtunkhwa, and Azad Jammu and Kashmir were selected on the bases of a lottery method. In the selected administrative units, 3 universities from each selected province (thus resulting in 12 universities and 84 respondents from each university) were selected on the bases of the lottery method. The lottery method is a technique used in simple random sampling that is a sampling method of probability sampling. In this method, all the population list is assigned a number and the numbers are then put in a bowl or hat, and then the numbers are selected from the hat until the desired number of sampling elements is received (48, 49). From these selected universities, four faculties were decided and out of these four faculties, respondents were conveniently approached (*n* = 21). The participants included both male (*n* = 531) and female (*n* = 477) university teachers with the age range of 26–60 years and *M* = 36.22, SD = 7.36. The sample included lecturers (*n* = 596), assistant professors (*n* = 338), and associate professors or above (*n* = 74). Only regular faculty members working in the current university for at least last 1 year were included in the sample. The employees who were not on regular bases or whose work experience in their current workplace was lesser than 1 year were not included in the sample.

### Instruments

The following instruments were used for this study.

### DUWAS-10 (1)

DUWAS-10 is a psychometrically sound measure of workaholism. It consists of 10 items that measure two dimensions of workaholism including Working Excessively and Working Compulsively. All the items are to be responded to on a four-point Likert Scale ranging from 1 = very unlikely to 4 = Always. For the current study, the Urdu-translated version (50) of the scale was used. The alpha reliability of the sub-scales of the Urdu version was acceptable i.e., 65 and 0.63 and 0.74 for Working Excessively, Working Compulsively, and total DUWAS-10 respectively.

### Oldenburg burnout inventory, OLBI (2)

To measure the level of burnout, the Urdu version (39) of OLBI (2) was used. It is a sound and reliable measure of burnout. It measures two dimensions of burnout including exhaustion and depersonalization through 16 items. All items are to be responded to on a four-point Likert scale where responses range from 1 = strongly agree to 4 = strongly disagree and half of the items are reversely coded. The scale is a reliable measure with high alpha i.e., 0.78 and 0.79 for Exhaustion and Disengagement respectively (51).

### Challenging job demands scale (3)

The Challenging Job Demands Scale (3) was used. It includes 13 items that measure three of the challenging job demands including Time Pressure (4 items), Social Load (5 items), and Cognitive Demands (4 items). The scale is a sound measure of challenging job demands with good psychometrics as the reported alpha is 0.81, 0.74, and 0.79 for Time Pressure, Social Load, and Cognitive Demands respectively.

### Anila PsyCap scale (4)

The level of PsyCap was measured by using Anila PsyCap Scale (4). The scale is a good measure of general psychological capital. The scale consists of 34 items categorized into 4 sub-scales i.e., Resilience, Self-Efficacy, Hope, and Optimism. The response format of the scale ranges from 1 to 4 where 1 indicates strongly disagree and 4 indicates strongly agree. The score is obtained by reverse coding item numbers 29 and 33 (which are reverse items) and then adding up the scores of all items. The scale is in the Urdu language and yields good psychometrics ranging from 0.67 (for the Hope sub-scale) to 0.87 for the total scale (4).

### Procedure

The present study was carried out from 2017 to 2019 and the data collection was completed by 2018. A research team was hired for data collection. Afterward, formal permissions of selected institutes were obtained. The sample was personally contacted by the researcher and their team who contacted the teachers in their offices and briefed them about the nature of the research. The study followed the ethical research protocol and key principles, for example, de-briefing about study objectives, voluntary participation informed consent (verbal and written), and confidentiality assurance. It was made clear to them that the research was non-funded and thus they would be given no incentive for participation in the research. They were further informed about their right to withdraw from the study at any point without facing any negative consequences for making such a decision before data collection. After having consent from the teachers, the questionnaires along with the demographic sheets were distributed among them. Some of them responded in the same meeting while some others gave time on another day. The researchers and the team contacted them at their promised time and collected the completed response. When the proposed number of the data was achieved, the data was thoroughly reviewed for missing items and face validity. 30 forms were having incomplete responses or random responses from 2 universes. These 30 forms were discarded and again 30 individuals from these universities were contacted thus, a total of 1,008 numbers were completed and were subjected to further statistical analyses.

### Statistical analysis plan

The frequency of all the variables was computed to identify the erroneous entry, missing values, and other initial information. After initial screening, Harman's Single Factor Test was applied to the data in which the researchers performed an EFA where all the items of the four scales were loaded on a single factor without rotation. The EFA revealed that only 15% variance of the single factor could explain which was quite acceptable to move further for the analysis assuming that the common method bias is not affecting the data (52). Afterward, Cronbach alpha reliability, mean and standard deviation, and correlations were computed by using SPSS as preliminary analyses so that the data may proceed to main analyses for hypothesis testing. The reliability analyses indicated that the data obtained was internally consistent enough to proceed with further analyses. The correlational analysis highlighted the initial pattern of relationship among study variables. Further, simple mediation was computed using Model 4 of Process Macro. Model 4 of Process Macro indicates the direct effect of an independent variable on an outcome variable with one or more mediators. In the present study workaholism was a predictor, job burnout was the outcome variable and the PsyCap was studied as a mediator. Further, the moderated mediation was carried out by using Model 14 of Process Macro. For the present study, the mediated relationship between workaholism and job burnout through PsyCap was studied at different levels of challenging job demands including time pressure, cognitive demands, and social load through this model.

### Ethical consideration

The Institutional Research Board, and Department of Psychology at the University of Sargodha, Pakistan (SU/PSY/786-3) initially approved the study. Afterward, the researcher hired a team for data collection who were initially trained about the ethical protocol suggested by the “Declaration of Helsinki” (respecting the dignity and autonomy of the participants; maintaining the confidentiality and all other rights of the participants including the right to refuse taking part in the study or withdraw their given information at any time).

## Results

To achieve the study objectives, the data obtained were subjected to analysis by using SPSS. Initially, Cronbach alpha reliability, mean and standard deviation, and correlations were computed by using SPSS. Further, simple mediation was computed using AMOS separately whereas, moderated mediation was computed using Model 14 of Process Macro. The results obtained are summarized below.

Table 1 summarizes the results of correlation among all the study variables. The results show that workaholism is negatively associated with burnout while it is positively associated with the three challenging job demands as well as with psychological capital. Psychological capital is negatively associated with burnout whereas, among job demands, only cognitive demands are negatively associated with burnout.

**Table 1:** Correlation matrix, psychometric properties and descriptive statistics of the scales used in the study.

**Variables**	**Workaholism**	**Job** **burnout**	**Time** **pressure**	**Cognitive** **demands**	**Social** **load**	**PsyCap**	***M***	**SD**	**α**
Workaholism	–	−0.17**	0.29***	0.12*	0.12*	0.28***	26.92	4.54	0.76
Job burnout	–	–	0.06	−0.14*	0.01	−0.41***	34.68	4.85	0.71
Time pressure	–	–	–	0.28***	0.23***	0.10	13.99	3.38	0.88
Cognitive demands	–	–	–	–	0.46***	0.20***	18.14	4.00	0.87
Social load	–	–	–	–	–	0.15*	13.43	3.15	0.70
PsyCap	–	–	–	–	–	–	106.71	10.43	0.91

*p < 0.05, ***p < 0.01, and ***p < 0.001.

Table 2 summarizes the results of the mediated relationship between workaholism and burnout through PsyCap. The results reveal that workaholism is a positive predictor of PsyCap (*B* = 0.64, *t* = 5.28, *p* < 0.001). Further, PsyCap (*B* = −0.18, *t* = −7.46, *p* < 0.001) negatively predicts burnout. The Path C describes total effect of workaholism on burnout which is (*B* = −0.16, *p* < 0.001) and the direct effects of burnout on workaholism is significant (*B* = −0.08, *p* < 0.05). Finally, the indirect effect of workaholism on burnout through PsyCap is significant i.e., (*B* = −0.15, *p* < 0.001; BootCILL = −0.19, BootCIUL = −0.11).

**Table 2:** Mediating role of PsyCap in the relationship of workaholism and burnout.

**Paths**	**Outcome variable**	**Predictor variable**	***B***	***P***	**95% CI**	
					**LL**	**UL**
A	PsyCap	Workaholism	0.76	0.000	0.62	0.90
B	Burnout	PsyCap	−0.20	0.000	−0.22	−0.17
C (Total effect)	Burnout	Workaholism	−0.08	0.019	−0.14	−0.01
C' (Direct effect)	Burnout	Workaholism	−0.15	0.000	−0.19	−0.11

**Figure 2:**
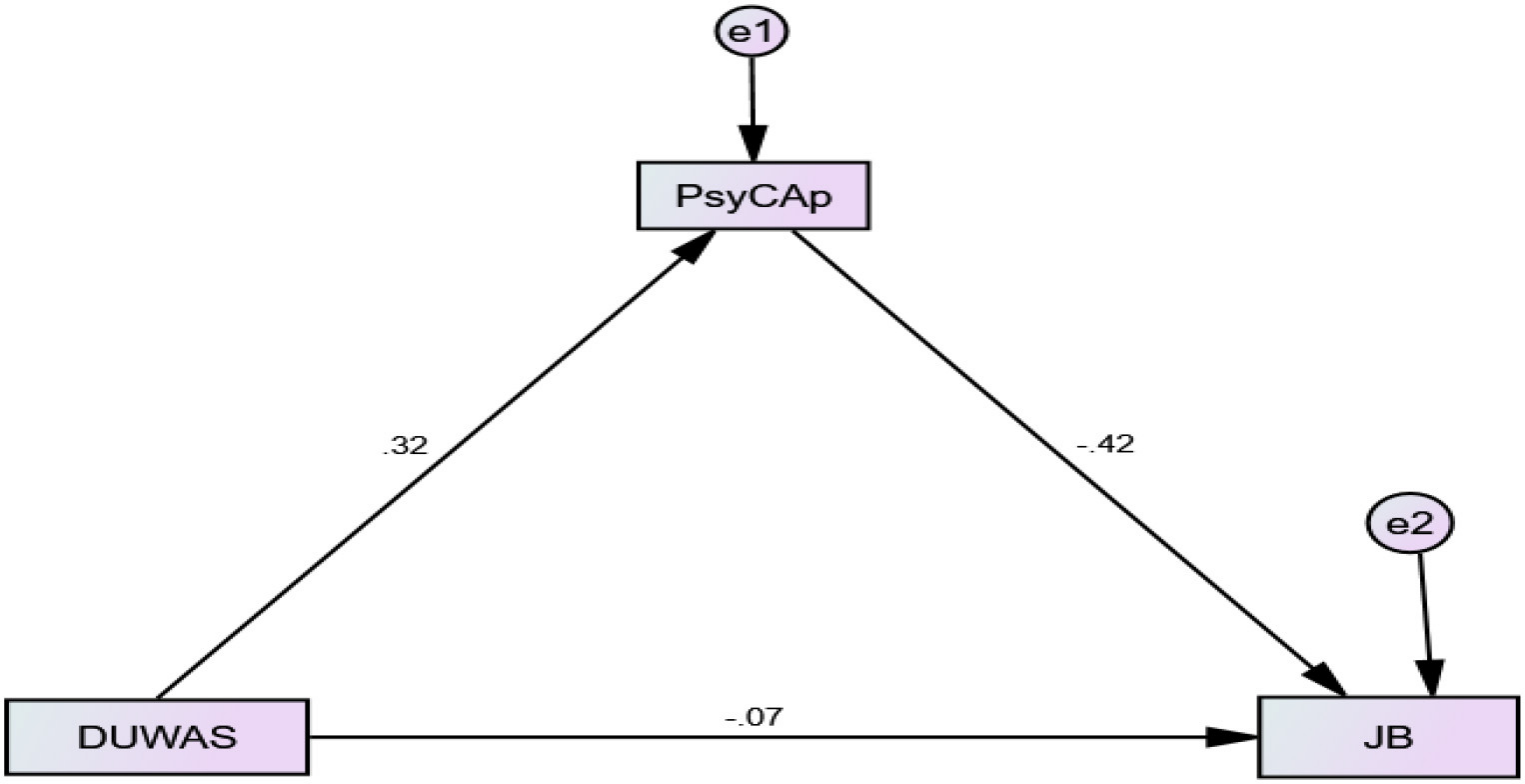
Mediated Relationship of Workaholism and Job Burnout through PsyCap.

Table 3 summarizes the results of moderated mediation analysis where PsyCap is studied as a mediator between the relationship between workaholism and job burnout. Moreover, the mediated relationship is moderated by time pressure, social load, and cognitive demands which moderate the relationship between PsyCap and job burnout. The index of moderated mediation states that the moderated mediation analysis for the moderating effects of time pressure (i.e., Index = −0.01, BootCILL = −0.02, BootCIUL = −0.004) and cognitive demands (Index = −0.01, BootCILL = −0.03, BootCIUL = −0.01) are significant (Index = −0.01, BootCILL = −0.02, BootCIUL = −0.004). Whereas, the social load is not a significant moderator for the mediated relationship of workaholism and burnout through PsyCap. Figure 2 gives a graphical representation of the results.

**Table 3:** Relationship of workaholism and job burnout mediated by PsyCap and moderated by time pressure, cognitive demands and social load (*N* = 1,008).

**Relationship of workaholism and job burnout** **mediated by PsyCap and moderated by time** **pressure**					**Relationship of workaholism and job burnout** **mediated by PsyCap and moderated by cognitive** **demands**					**Relationship of workaholism and job burnout** **mediated by PsyCap and moderated by social load**				
**95% CI**					**95% CI**					**95% CI**			
**Paths**	***B***	***LL***	***UL***	***P***	**Paths**	***B***	***LL***	***UL***	***p***	**Paths**	***B***	***LL***	***UL***	***P***
Workaholism → PsyCap	0.76	0.62	0.90	0.000	Workaholism → PsyCap	0.76	0.62	0.90	0.000	Workaholism → PsyCap	0.76	0.62	0.90	0.000
PsyCap →	0.02	−0.10	0.15	0.71	PsyCap → JB	0.11	−0.02	0.25	0.092	PsyCap → JB	−0.08	−0.21	0.06	0.069
TP → JB	1.63	0.73	2.53	0.000	CD → JB	1.44	0.81	2.06	0.000	SL → JB	0.74	0.024	1.46	0.042
WKh → JB (Direct effect)	−0.10	−0.16	−0.03	0.006	WKh → JB (Direct effect)	−0.05	−0.16	0.06	0.394	WKh → JB (Direct effect)	−0.09	−0.15	−0.02	0.010
Workaholism → PsyCap → JB^a^	−0.12	−0.17	−0.07	0.000	Workaholism → PsyCap → JB^a^	−0.10	−0.15	−0.07	0.000	Workaholism → PsyCap → JB^a^	−0.17	−0.21	−0.12	0.000
Workaholism → PsyCap → JB^b^	−0.19	−0.22	−0.17	0.000	Workaholism → PsyCap → JB^b^	−0.19	−0.22	−0.17	0.000	Workaholism → PsyCap → JB^b^	−0.20	−0.22	−0.17	0.000
Workaholism → PsyCap → JB^c^	−0.22	−0.26	−0.19	0.000	Workaholism → PsyCap → JB^c^	−0.24	−0.27	−0.20	0.000	Workaholism → PsyCap → JB^c^	−0.22	−0.26	−0.19	0.000
PsyCap × TP	−0.01	−0.02	−0.01	0.000	PsyCap × CD	−0.01	−02	−0.01	0.000	PsyCap × SL	−0.01	−0.03	0.01	0.369

JB, Job Burnout; TP, time pressure; PsyCap, Psychological capital; CD, cognitive demands; SL, social load. The relationship of WKH and JB through PsyCap at ^a^low level of moderator; ^b^moderate level of moderator; ^c^at high level of moderator.

The Table 3 states that workaholism significantly and positively predicts PsyCap (*B* = 0.65, *t* = 5.22, *p* < 0.0001); however, in this model, PsyCap doesn't predict job burnout. Moreover, time pressure predicts burnout positively (*B* = 2.01, *t* = 2.42, *p* < 0.05). The interaction term suggests that PsyCap and time pressure interact with each other and produce a significant effect on the outcome variable (*B* = −0.02, *t* = −2.21, *p* < 0.05). Further, the direct path indicates that workaholism does not predict job burnout directly whereas, the indirect effects on the low, moderate, and high levels of time pressure are significant. Further, when the moderating role of the social load is observed, the table indicates that all the direct paths to burnout are non-significant except the direct and indirect effects of workaholism suggesting that workaholism is a significant predictor of burnout and PsyCap. Whereas, PsyCap, social load, and workaholism do not predict job burnout. However, the mediated relationship of workaholism and burnout through PsyCap on a low, moderate, and high level of moderators is significant (with negligible difference in the value of B for all the three levels of the moderator) suggesting that mediation is significant but the moderated mediation is non-significant.

Finally, when role of cognitive demands is observed, it is turned out that workaholism significantly predicts PsyCap (*B* = 0.72, *t* = 5.72, *p* < 0.0001) and cognitive demands significantly predict burnout (*B* = 1.94, *t* = 2.765, *p* < 0.05); whereas, workaholism and PsyCap are non-significant predictors of the burnout. Further, the results revealed that the interaction of PsyCap and cognitive demands produce significant effects on job burnout with ΔF_(1, 323)_ = 8.56, *p* < 0.01 and contributes to a 2.09% variance in the outcome variable (ΔR2 = 0.0209). The pictorial representation of the significantly mediated moderations is given in the figures.

**Figure 3:**
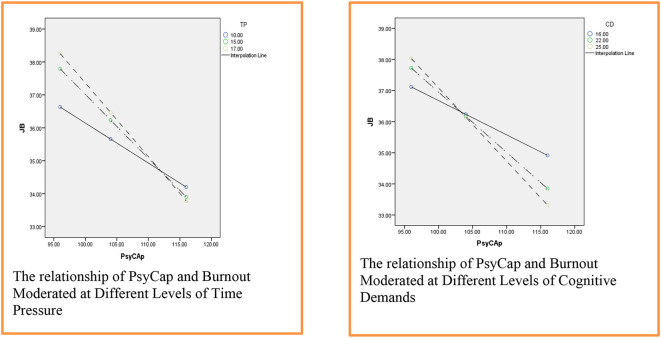
The relationship of PsyCap and burnout moderated at different levels of time pressure, cognitive demands and social load (1,008).

The Figure 3 gives a clearer look at the moderated relationship between PsyCap and burnout where challenging job demands are supposed to be moderators.

**Figure 4:**
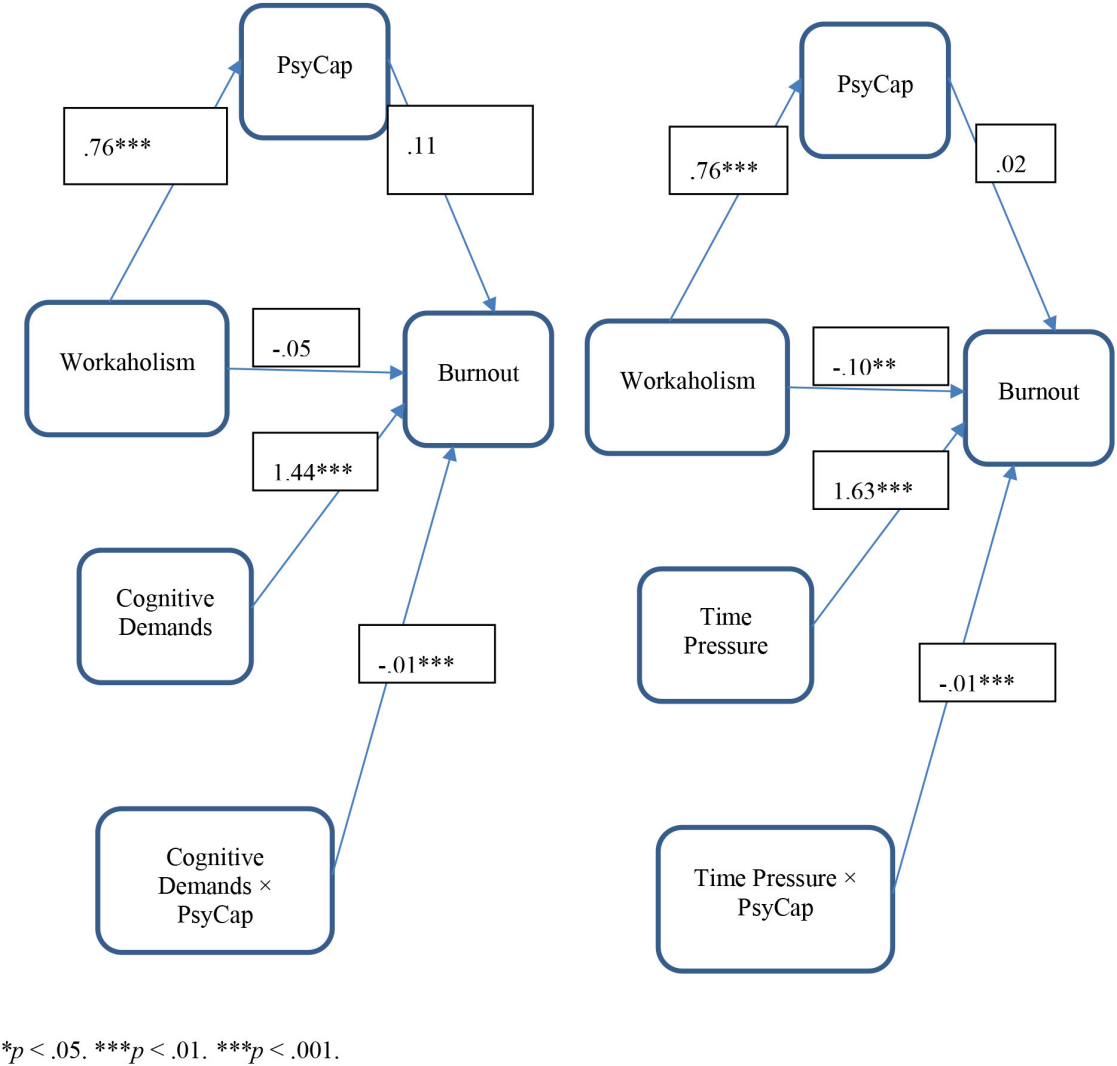
Relationship of workaholism and job burnout mediated by PsyCap and moderated by time pressure and cognitive demands (*N* = 1,008).

Figure 4 illustrates the moderated relationship between PsyCap and burnout at different levels of challenging job demands. The figure states that time pressure and cognitive demands both act as challenging in the relationship of independent and criterion variables such that the higher the level of challenging job demand (i.e., time pressure and cognitive demands) the stronger the relationship of PsyCap and job burnout. However, the social load does not moderate the relationship between the two.

## Discussion

The study was aimed at examining the mediated relationship between workaholism and burnout through PsyCap at different levels of challenging job demands including time pressure, social load, and cognitive demands. The results revealed that workaholism significantly and positively predicted PsyCap which negatively predicted burnout. The total direct and indirect effects of workaholism on job burnout were found significant suggesting a mediation effect.

The present study is an endeavor to find out the status of workaholism in the JD-R Model. Within JD-R Model, the status of workaholism is controversial to some researchers (53). Traditionally, workaholism is found a negative predictor of work-related outcomes more specifically that of occupational health and burnout. However, there is more to know about the exact role of workaholism in the JD-R Model (54). Not only is it related to burnout, but also has a strong negative relationship with quality of life (39). Similarly, an increase in excessive working (a dimension of workaholism) is associated with an increase in sick leave as well as burnout (55). However, it may produce positive effects too. For instance, simply giving more time to work and thinking more about work may bring more work done. Similarly, it may yield more positive ratings by the supervisors as compared to those for non-workaholics. Moreover, they want to avoid negative emotions which arise for them when not working, and thus gain more satisfaction from work (56). Therefore, their work may bring more positive experiences for them such as hope, resilience, efficacy, and optimism, i.e., the higher PsyCap. This enhanced PsyCap eventually results in decreased burnout. Previous research has established this relationship (57) by stating that PsyCap results in enhanced well-being as those with high PsyCap are more able to experience flourishing as well as are more engaged, which, in turn, reduces their burnout. Previous studies (58, 59) also asserted PsyCap as a mediator between the relationship between workaholism and burnout and other constructs in JD-R Model.

Further, two of the challenging job demands (i.e., time pressure and social load) moderated the mediated relationship in the sense that mediated relationship was stronger with the higher level of challenging job demands (H2) however, the social load did not moderate the relationship. Traditionally, psychological capital had been observed as a strong negative predictor of burnout (35, 60, 61) and as the present study concluded, the negative relationship was stronger for those who experience challenging job demands at work. This stance of challenging job demands is not new to JD-R Model as challenging job demands have previously been observed as moderators in the literature of the JD-R Model (62, 63). Since challenging job demands may foster psychological need satisfaction and thus may bring important health-related and occupational benefits (64), it may eventually result in reduced burnout. Moreover, those high on PsyCap when facing challenging job demands may take more benefits from their demands and thus ultimately may experience lesser burnout. Therefore, the moderating relationship of challenging job demands seems justified. However, the social load did not moderate the stated relationship. One possible factor behind this may lie in the multi-dimensional nature of social relationships at work, particularly at universities. Some social interactions need cognitive efforts while others may end up only in fatigue. Hence, a piecemeal treatment of social load is further required.

Earlier theories and research are controversial regarding the effects of time pressure on burnout. For instance, some researchers (65, 66) found a moderate to a strong positive relationship between time pressure with the dimensions of burnout. However, some others (67) found that time pressure resulted in different types of creative behaviors and outcomes based on positive and negative affective states. The results of these studies suggest that the effects of time pressure on different attitudes and behaviors are not simple but the result of the interaction of other variables.

As in the present case, the results revealed that time pressure itself produces a significant (although weak) positive effect on job burnout. However, the negative effects on burnout are observed when the employees possess more PsyCap and face high time pressure. In other words, workaholics who possess a high level of self-efficacy, hope, optimism, and self-efficacy experience a lesser level of burnout in situations where they face high time pressure. Stating simply, being a workaholic alone does not affect burnout. Rather, it is a matter of personal resources as well as the demands from the side of the organization (in this case time pressure) which lead the workaholic toward the experience of lesser or more symptoms of burnout.

The results supported cognitive demands as a moderator in the mediated relationship between workaholism and burnout. Stating otherwise, workaholics who had more psychological strength in terms of resilience, hope, self-efficacy, and optimism were less likely to experience burnout when they found that their jobs were offering them a means of personal development and grooming (i.e., high cognitive demands). Moreover, cognitive demands serve as a means to fulfill the need for competence. This need satisfaction might have also resulted in decreased burnout. The previous research (68) noted that PsyCap helps in need fulfillment (including the need for autonomy, competence, and relatedness) which in turn leads to better psychological well–being and enhanced performance.

The next challenging job demand was a social load which did not moderate the stated mediated relationship (see Table 3). The social load includes the number of contact with others at work for work-related tasks. This interaction results in both positive and negative consequences for the employees (3, 40). Besides being a source of broader exposure to problem-solving, a means to satisfy relatedness needs, and a source of personal recognition at work, the social load may result in potentially negative consequences. For instance, it gives room to high emotional demands and emotional labor.

Emotional labor and emotional demands are required in professions where positive or negative emotions are required in order to complete the task successfully. More specifically, emotional labor is the requirement for service professionals who have to show (no matter what their inner emotional state is) positive emotions at work (69). Therefore, in service-oriented professions, contact with others at work demands emotional labor from them as the employees have to display the required and not the original emotions. Consequently, the professionals might experience negative outcomes. Keeping both positive and negative outcomes together, the social load might have worked both as a challenge and hindrance stressor as well. A piecemeal analysis of social load might produce different results.

To conclude, the relationship between workaholism and burnout was mediated through PsyCap and the challenging job demands (including time pressure and cognitive demands but not social load) moderated the relationship. The study yields important implications for organizational researchers who can replicate the findings as well as can study the role of more personal resources and other challenging job demands. Further, it is important for the organizational leaders and managers, who can foster the health conditions of the employees by offering more challenging job demands at work and by organizing workshops based on PsyCap to employees specifically those who are high on workaholism to prevent them from burnout and other health-related issues.

### Limitations of the present study

The study has some limitations. For example, the sample included more lecturers and assistant professors and a lesser number of higher-level university teachers. Although this is because of the actual trend of teachers in Pakistani universities where teachers of higher positions are lesser in numbers, this may act as confounding. Besides, workaholism, burnout, and social load are all multi-dimensional variables but the research did not assess the relationships on a finer-grained level due to time constraints. Therefore, future studies may replicate the findings by searching for and/or using the sub-scales of the constructs. Finally, the study has focused only on the health impairment process, however, the same positive effects of personal resources and challenging job demands can be explored in the process of work engagement.

### Theoretical and practical strengths of the study

At the theoretical level, the study adds to the literature on JD-R Model in the following ways:

The study adds to the positive side of workaholism, hence it does not attribute “goodness” or “badness” inherent to workaholism itself; rather it offers the underlying mechanisms that make workaholism “good” or “bad.” Although personal resources such as psychological capital have traditionally been studied as predictors or moderating factors; the study adds to the literature on the JD-R Model by suggesting PsyCap as a mediator. It also contributes to the validation of the JD-R Model by confirming the interactive role of challenging job demands, which had been established by previous theorists and researchers working with this theory of occupational health and well–being.

The study offers important implications to the administration of the universities as well as to the occupational health counselors. It suggests that the administrators can specifically target the workaholics and foster their psychological capital through different faculty development workshops. Afterward, challenging job demands may be assigned to them which will even bring more positive outcomes regarding the level of burnout. Further, the study offers the same to the workaholics themselves who can work on their personal resources to beget more benefits from their workaholism.

## Conclusions

Burnout is an occupational health problem that not only affects the employees' health but also harms the productivity and performance of the organization. This results in devastating effects on the economy of the organization. In the present world, workaholism, or the addiction to work is also a serious health concern. However, working on personal resources may affect the relationship between workaholism and burnout in higher education institutes.

## Data availability statement

The original contributions presented in the study are included in the article/supplementary material, further inquiries can be directed to the corresponding authors.

## Ethics statement

The studies involving human participants were reviewed and approved by the Institutional Research Board, and Department of Psychology at the University of Sargodha, Pakistan (SU/PSY/786-3) initially approved the study. The patients/participants provided their written informed consent to participate in this study.

## Author contributions

The study is designed by IM, NIM, and MA. While MS, NM, MQ, and KT gave the creative contribution to the finalization of this manuscript. All the authors have equally contributed to the write-up, critical review, and finalization of the manuscript. All authors contributed to the article and approved the submitted version.

## Conflict of interest

The authors declare that the research was conducted in the absence of any commercial or financial relationships that could be construed as a potential conflict of interest.

## Publisher's note

All claims expressed in this article are solely those of the authors and do not necessarily represent those of their affiliated organizations, or those of the publisher, the editors and the reviewers. Any product that may be evaluated in this article, or claim that may be made by its manufacturer, is not guaranteed or endorsed by the publisher.
